# Supplementary material for: An Atypical ABC Transporter Is Involved in Antifungal Resistance and Host Interactions in the Pathogenic Fungus Cryptococcus neoformans
Source: mBio. 2022 Jun 21;13(4):e01539-22. doi: 10.1128/mbio.01539-22 (PMC9426558; doi:10.1128/mbio.01539-22)
Supplement: TEXT S1 [file mbio.01539-22-s0001.docx]

**Supplemental Text S1**

Winski CJ *et al*. An atypical ABC transporter is involved in antifungal resistance and host interactions in the pathogenic fungus *Cryptococcus neoformans*

**Fungal strains, growth conditions, and reagents**

All *C. neoformans* strains used were in the serotype A strain H99α. H99α *afr1*Δ and H99α *afr1*Δ/*afr2*Δ/*mdr1*Δ were a generous gift from Yun Chang (National Institutes of Health)(1). The *S. cerevisiae* strain ADΔΔ and the plasmids pABC3 and pABC3-XLmGH were graciously shared by Richard Cannon (Otago University, New Zealand)(2). All fungal strains were maintained at -80ºC and grown at 30ºC on yeast peptone dextrose (YPD) with antibiotics as appropriate, or minimal complete media (YNB) with the appropriate nutritional selection.

Fluconazole (FLC; VWR), amphotericin B (AMB; VWR), caspofungin (CSF; Sigma), ketoconazole (KTC; Sigma), itraconazole (ITC; Sigma), voriconazole (VRC; Sigma), posaconazole (PSC; Sigma), nocodazole (Sigma), berberine (BER; Sigma), and trichostatin A (TSA; VWR) were prepared in dimethyl sulfoxide (DMSO) at 200 mg/mL, 20 mg/mL, 5 mg/mL, 2 mg/mL, 4 mg/mL, 25 mg/mL, 2 mg/mL, 2 mg/mL, 2 mg/mL, 20 mg/mL, and 1 mg/mL, respectively. Paraquat (PQ; Sigma) and 5-flucytosine (5-FC; Sigma) were dissolved in sterile distilled water at 20 mg/mL and 5 mg/mL. Menadione was dissolved in ethanol at 10 mM. Stock solutions were kept at -20ºC.

**Naming of *PDR6* (CNAG_06909) and other PDR genes**

We used the prototypical *S. cerevisiae* Pdr5 protein sequence to search the genomes of all 21 fungal species depicted in Figure 1A. We were using strict parameters, including 80% coverage and BLASTp E-values of at least 1 x 10^-15^. We found 10 genes in *C. neoformans* strains H99 and KN99, the most common lab reference strains, that meet these criteria (see Table 1 below). 5 of those 10 genes had been described, or named, previously: *AFR1* (in 2006), and *SNQ1*, *PDR5*, *PDR5-2*, and *PDR5-3* (in 2009). We sorted the remaining 5 genes by ORF number and named them *PDR2*, *PDR4*, *PDR6*, *PDR7*, and *PDR9* following the nomenclature guidelines in the field (3) that call for using gene names that are not already used in *S. cerevisiae* when the two genes are not clear homologues, which is the case here.

Table 1. PDR-type ABC transporters in *C. neoformans*^a^

| **Gene ID** | **Name (Alias)** | **Size (amino acids)** | **Orthologue(s) in *S. cerevisiae*^b^** | **Clade (Fig. 1B)** | **Reference(s)^c^** |
| --- | --- | --- | --- | --- | --- |
| CNAG_00730 | *AFR1* | 1543 | *SNQ2, PDR18, PDR5, PDR12* | III | Sanguinetti M et al. (2006) |
| CNAG_06338 | SNQ1 | 1420 | *PDR18, PDR15, PDR5, SNQ2, PDR10* | IV | Ko YJ et al. (2009) |
| CNAG_00869 | *PDR5* (*AFR2*) | 1529 | *PDR15, PDR5, PDR10, SNQ2, PDR12, PDR18* | I | Ko YJ et al. (2009)  Basso LR Jr et al. (2015) |
| CNAG_04098 | *PDR5-2* | 1536 | *PDR15, PDR5, PDR10, SNQ2, PDR12, PDR18* | I | Ko YJ et al. (2009) |
| CNAG_06348 | *PDR5-3* | 1421 | *PDR15, PDR18, PDR5, SNQ2, PDR12* | IV | Ko YJ et al. (2009) |
| CNAG_05470 | *PDR2* | 1241 | YOL075C | VIa |  |
| CNAG_06533 | *PDR4* | 1173 | *ADP1* | V |  |
| CNAG_06909 | *PDR6* (*AFR3*) | 626 | None | VIb | This study  Oliveira NK et al. (2022)^d^ |
| CNAG_07619 | *PDR7* | 560 (t1)^e^  544 (t2) | None | I |  |
| CNAG_07799 | *PDR9* | 1462 | *SNQ2, PDR18, PDR12, PDR5, PDR15, PDR10* | III |  |

^a^ In the genome of reference lab strains H99 and KN99.

^b^ Shown are the *S. cerevisiae* genes with a BLASTp E-value of “zero” when compared to the *C. neoformans* protein database. In cases where there is a reciprocal best match, one gene name is given. If no protein had a coverage of > 80% and an E-value < 10^-15^, then “None” is shown.

^c^ The reference of the original paper describing the gene is given. Sometimes the same gene was given two different names, in those cases both references are given.

^d^ At the time this study was submitted for publication, Oliveira NK et al. was available only as a preprint (doi: 10.20944/preprints202201.0263.v1).

^e^ This gene has two transcripts annotated in the FungiDB database.

**Fungal genome manipulation**

We tried on several occasions to complement the *pdr6*Δ strain on the original locus unsuccessfully. The few colonies obtained by biolistic transformation did not have the *PDR6* construct integrated correctly; although some of these randomly integrated complemented strains restored antifungal susceptibility, it did not fully restore other phenotypes. Since complementation proved challenging, and it is as time-consuming as creating deletions, we used the split marker method (4) to delete *PDR6* in H99α. We used genomic DNA from the strain 1F1 (*pdr6*Δ) from the Madhani deletion collection (obtained from the Fungal Genetics Stock Center, <http://www.fgsc.net/>) as the template to amplify the NAT resistance split marker fragments for the biolistic. Information on the primers to use for each gene in the deletion collection is provided by the Fungal Genetics Stock Center. Three independent *pdr6*Δ mutant strains were generated and confirmed to be correct by PCR. These recreated strains were used to verify that all observed phenotypes are a direct result of the loss of *PDR6* (see Fig. S1, S2, 8, and 9). Additionally, we performed whole genome sequencing on generated *pdr6*Δ mutants to confirm proper genetic manipulation of the strains (to ensure that indeed the gene was gone without alterations to flanking regions).

**Tissue culture cells, growth conditions, and reagents**

The human monocyte cell line THP-1 (ATCC TIB-202) was grown in THP-1 complete media (RPMI-1640 with L-glutamine supplemented with 1mM sodium pyruvate, 0.05% 2-mercaptoethanol, 10% FBS, and 100 units/mL Penicillin – 100μg/mL Streptomycin solution) and differentiated with phorbol 12-myristate 12-acetate (PMA, from Sigma, St. Louis, MO) as described in (5). THP-1 cells were split every 3-4 days and new vials were thawed every month.

**Drug-susceptibility assays**

The CLSI M27-A3 reference method was used to determine the MIC of each drug (40). Briefly, compounds were dispensed in a 96-well plate with 2-fold serial dilutions. The final concentrations were prepared from stock solutions in RPMI 1640 medium. The concentration ranges tested were as follows: FLC, CHX, 8 – 0.03125 μg/mL; AMB, BER, 16 – 0.0625 μg/mL; nocodazole, ITC, VRC, KTC, PSC, 1 – 0.00390625 μg/mL; PQ, 100 μg/mL – 0.390625 μg/mL; TSA, 64 – 0.25 μg/mL. 500 cells were spotted in each well from overnight cultures. Plates were incubated at 37ºC + 5% CO_2_ for 48 – 72 hr. Minimal inhibitory concentrations (MICs) were quantified by measuring the OD_600_ using a microtiter plate reader.

The MIC of FLC was also determined on RPMI plates with Epsilometer test strips (Etest strips; Liofilchem Thermo). Approximately 5 X 10^4^ cells were plated on RPMI agar plates before application of the E-test strips. Plates were incubated at 30ºC for 48 hr.

**Checkerboard assays**

Checkerboard assays to assess the fractional inhibitory concentrations (FICs) for combinations of antifungal drugs was performed as previously described (1). Briefly, strains to be tested were grown overnight in YPD, diluted to OD_600_ of 0.2, and grown for two doublings. The cultures were diluted in RPMI 1640 to 100 cells/μl. All antifungal stocks were diluted in RPMI 1640 to the following final concentration ranges: FLC, 32 to 0.25 μg/mL; AMB, 4 to 0.0625 μg/mL; CSF, 16 to 0.125 μg/mL; and 5-FC, 16 to 0.125 μg/mL. Assays were incubated at 37ºC + 5% CO_2_. FIC index (FICI) values for a combination of compound A and B were calculated as:

$$\mathrm{FICI}= \frac{{MIC}_{A} in combination}{{MIC}_{A} alone} + \frac{{MIC}_{B} in combination}{{MIC}_{B} alone}$$

Where a FICI value of ≤ 0.5 is considered synergistic, additive if the value is between 1.0 and 0.5, and antagonistic if the FICI is ≥ 2.0.

**Synthesis of Probe 1**

**General Methods:** Unless otherwise specified, commercially available organic reagents and solvents were used without further purification. Reactions progress was monitored by analytical TLC analysis (pre-coated silica-gel aluminum foils, 60 F_254_, Merck KGaA, Darmstadt, German) under 254 nm UV light. The crude products were purified by a Combi-flash Rf chromatography system (Teledyne Technologies, Inc., Thousand Oaks, CA). NMR spectra (^1^H, ^13^C and ^19^F) were performed on a Bruker AVANCE Ⅲ HD 400 Nanobay (400 MHz for ^1^H, 101 MHz for ^13^C and 376 MHz for ^19^F, Bruker Biospin AG, Fällanden, France) at 25 ºC and the residual non-deuterated solvent signals were used as reference (chloroform-*d*, ^1^H δ = 7.26 ppm, ^13^C δ = 77.2 ppm). High-resolution mass spectra (HRMS) were performed on a Bruker micrOTOF/Q2 mass spectrometer (Bruker Daltonics Inc., Fremont, CA) with positive electrospray ionization (ESI^+^). The purity of the final compounds was analyzed by a Dionex Ultimate 3000 HPLC system (Thermo Fisher Scientific Inc., Waltham, MA) with an Acclaim™ RSCL 120 C18 column (0.2 µm, 120 Å, 2.1 × 100 mm, Thermo Fisher Scientific Inc., Waltham, MA). All the final compounds were determined to be greater than 95% pure.

**Synthetic procedures for *N*-(2-(2,4-difluorophenyl)-2-hydroxy-3-(1*H*-1,2,4-triazol-1-yl)propyl)-5-(dimethylamino)naphthalene-1-sulfonamide** **(Probe 1):** 1-Amino-2-(2,4-difluorophenyl)-3-(1H-1,2,4-triazol-1-yl)propan-2-ol (52 mg, 0.20 mmol), synthesized pursuant to the reported procedure (6), and trimethylamine (29 μL, 0.20 mmol) were added in anhydrous dichloromethane (5 mL), followed by the addition of dansyl chloride (55 mg, 0.20 mmol). The resulting solution was stirred at room temperature for 24 h. After the completion of the reaction, the solvent was removed under reduced pressure to give a yellow solid. The yellow solid was then purified by Combi-flash Rf chromatography system (DCM:MeOH = 99:1 to 97:3) giving a yellow oil. The oil was triturated with hexanes to afford the desired product as a yellow solid (34 mg, 34%). This procedure was adapted from a literature report (7). ^1^H NMR (400 MHz, CDCl_3_) δ 8.52 (d, *J* = 8.5 Hz, 1H), 8.17 (dd, *J* = 1.1, 7.3 Hz, 1H), 8.10 (d, *J* = 8.7 Hz, 1H), 7.86 (s, 1H), 7.75 (s, 1H), 7.54 – 7.40 (m, 2H), 7.35 – 7.26 (m, 1H), 7.15 (d, *J* = 7.6 Hz, 1H), 6.58 (dt, *J* = 2.4, 8.3 Hz, 1H), 6.48 (ddd, *J* = 12.0, 8.4, 2.5 Hz, 1H), 5.62 (t, *J* = 6.6 Hz, 1H), 5.16 (s, 1H), 4.66 (d, *J* = 14.4 Hz, 1H), 4.58 (d, *J* = 14.3 Hz, 1H), 3.42 (dd, *J* = 7.4, 13.7 Hz, 1H), 3.21 (dd, *J* = 5.9, 13.7 Hz, 1H), 2.87 (s, 6H). ^13^C NMR (101 MHz, CDCl_3_) δ 162.8 (dd, *J*_CF_ = 14.5, 253.0 Hz, 1C), 158.3 (dd, *J*_CF_ = 11.6, 245.8 Hz, 1C), 152.1, 152.0, 144.4, 134.1, 130.9, 130.1 (dd, *J*_CF_ = 5.6, 9.5 Hz, 1C), 130.0, 129.7, 129.4, 128.6, 123.2, 122.6 (dd, *J*_CF_ = 3.8, 12.9 Hz, 1C), 118.5, 115.4, 111.8 (dd, *J*_CF_ = 3.2, 20.8 Hz, 1C), 104.0 (t, *J*_CF_ = 26.6 Hz, 1C), 75.3 (d, *J*_CF_ = 4.7 Hz, 1C), 54.8 (d, *J*_CF_ = 5.8 Hz, 1C), 49.2 (d, *J*_CF_ = 4.2 Hz, 1C), 45.5. ^19^F NMR (376 MHz, CDCl_3_) -109.8 (d, *J* = 8.4 Hz, 1F), -109.4 (d, *J* = 8.4 Hz, 1F). HRMS-ESI (*m/z*): [M + H]^+^ calcd for C_23_H_24_F_2_N_5_O_3_S, 488.1562; found, 488.1567.

**Flow-cytometry analysis of the efflux of R6G, Nile Red, and Probe 1**

Accumulation assays were performed as previously described (8). Briefly, strains were grown overnight in YPD at 30ºC with shaking. Cultures were diluted to an OD_600_ of 0.5 in YPD. Cells were incubated in the presence of 10 μM R6G (VWR), 7 μM Nile Red (Thermo), or 10 μg/mL Probe 1 for 30 min at 30ºC with shaking. The reaction was stopped by cooling on ice. The mixture was diluted 40-fold in cold 1X PBS. The accumulation was immediately assessed by flow cytometry. R6G and Nile Red samples were excited with a 488 nm laser and a PE-Texas Red filter was used to detect mean fluorescent intensity (MFI) values. Probe 1 samples were excited with a 405 nm laser. For each sample, 10,000 events were collected and data was analyzed using FlowJo software.

**Liquid Chromatography/Mass Spectrometry (LC/MS)**

5 mL of log-phase culture were collected, washed, and resuspended in 1 mL of PBS. Then DMSO or the indicated FLC amounts were added and incubated at 30 °C with shaking for 30 minutes. At this point, 200 μL were removed for CFU quantification and the rest was spun down at 4 °C, washed with cold PBS, and resuspended in cold lysis buffer (50 mM Tris, pH 8, 150 mM NaCl). ½-volume of glass beads was added and the cell suspension was agitated in a beadbeater for 4 minutes in the cold. The cells were spun down and the lysate was transferred to a new tube were the same volume of cold MeOH was added. The solution was mixed and spun down at 4 °C at 21,300 x g for 10 minutes. The supernatant was transfered to a new tube and mixed with 1 volume of CH_2_Cl_2_, then vortex and spun as above. At this point the organic (bottom) phase was transferred into a glass tube with a gel-loading tip and evaporated with a speed-vac. The dried sample was resuspended in 100 μL of MeOH, mixed well prior to adding 400 μL of water. This crude sample was further cleaned by passing through a MCX (mixed-mode cationic exchange) SPE column following the manufacturer’s instructions. The elutes from SPE were concentrated by speedvac, reconstituted in 50:50 water:MeOH, and analyzed using LC/MS. The LC instrument consisted of a Waters Acquity UPLC H-Class system equipped with a photodiode array detector, a sample manager-FTN, and quaternary solvent manager. LC separations were accomplished on a Waters UPLC Acquity BEH C18 (2.1 50 mm, 1.7 μm) with a Waters UPLC Acquity BEH C18 VanGuard TM Pre-column (2.1 5 mm, 1.7 μm) at 50 °C with a 4-min isocratic at 80% A/20% B (A = 0.1% formic acid in water, B = 0.1% formic acid in MeOH) with a flow rate of 0.4 mL/min. LC flow during the first 1.5 min of each run was diverted to the waste. A Bruker impact II ultrahigh resolution Qq-time-of-flight mass spectrometer was coupled with the LC system described above using Hystar 5.0 SR1 software. The Bruker electrospray ionization source was operated in the positive ion mode with the following parameters: capillary voltage = 1800 V, end plate offset voltage = −500 V, and nitrogen as both a nebulizer (4 bar) and dry gas (7 L/min) at 200 °C (dry heater temperature). Mass spectra were accumulated over the mass range 150−3000 Da. Peak area from extracted-ion chromatogram of FLC (m/z 307.1113 as [M+H]^+^)was calculated using Bruker Compass DataAnalysis 5.1 software and used for quantification.

**Phenotyping**

Strains tested were grown overnight in YPD, diluted to an OD_600_ of 0.2, and grown for two doublings. The cultures were diluted to 1 x 10^7^ cells/mL and serially diluted (10-fold) and spotted (5 μL) onto YPD with 1 M NaCl, 0.005% SDS, 0.1% Congo Red, and 0.5 μg/mL caffeine. Plates were incubated at 30ºC for 48 – 72 hr.

Gradient plates were performed as previously described (9). Briefly, for preparation of FLC gradient plates, 20 mL of RPMI-agar are dispensed with a sterile glass pipet into petri dishes that are set on an angle such that the agar solution diminishes to nothing at the raised edge of the plate. The plate is kept inclined until the layer solidifies, at which point the plate is set leveled on a surface. At this point FLC is added to the molten RPMI-agar and 20 mL of the agar media with the FLC are dispensed on top of the solidified layer. Once solidified and dried, 500 cells in 5μL of the indicated strains were spotted across the plate from the side with no FLC to the side of highest FLC concentration.

**Uptake Assay**

Uptake assays were performed as previously described (10). Briefly, THP-1 cells were seeded in a 96-well plate and incubated for 48 hr at 37ºC + 5% CO_2_. Overnight cultures of *C. neoformans* strains were diluted to an OD_600_ of 0.2 and grown for two doublings. The log phase cultures were washed with PBS, incubated with Lucifer Yellow dye (Sigma) for 30 min, and opsonized with 40% human serum for 30 min. Serum was obtained from healthy donors with informed consent under a protocol approved by the University of Notre Dame Institutional Review Board. The assay was initiated by removing medium from wells containing THP-1 cells and the opsonized fungal cells were added. Following a 1 hr incubation, the plates were washed using a microplate washer (405LS, Biotek, Winooski, VT), fixed with 4% formaldehyde, and stained with DAPI (Sigma) and CellMask Deep Red (Invitrogen). NaN_3_ in PBS was added to the plates and images were obtained using a Zeiss microscope. Phagocytic index (PI) values were quantified using Cell Profiler software.

**Conditioned media**

Conditioned media (CM) was generated as previously described (11). Briefly, overnight cultures were diluted to 1 X 10^5^ cells/mL in RPMI 1640 media and incubated for 5 days at 30ºC. Cells were removed by centrifugation and the supernatant was filtered through a 0.2-μm filter.

**Capsule induction**

Cells were grown overnight in YPD, washed with DMEM, and 1 X 10^6^ cells/mL were added to 24-well tissue culture plates. Plates were incubated at 37ºC + 5% CO_2_ for 24 hr. Cell suspension was collected and washed, India ink was added, and samples were visualized on a Zeiss microscope. Images were analyzed with ImageJ (NIH) for capsule thickness.

**Capsule-shedding assay**

Capsule shedding assays were performed as previously described (12). Briefly, strains were grown overnight in YPD at 30ºC, washed, and diluted to 1 X 10^6^ cells/mL in RPMI 1640. Following a 15 hr incubation at 37ºC + 5% CO_2_, the samples were heated to denature enzymes, centrifuged to separate cells and supernatant, and then stored at 4ºC. The samples were loaded on a 0.6% certified megabase agarose (Bio-Rad) gel and subjected to electrophoresis for 15 hr at 25 V. Gel contents were transferred to a positively charged membrane using a standard Southern blot protocol with 10X SSC. Following overnight transfer, the membrane was blocked for 48 hr in 1X TBS-5% milk and incubated for 1 hr in 1X TBST-1% milk with anti-GXM monoclonal antibody at 1:10,000. Membrane was rinsed three times in 1X TBST and incubated for 1 hr in 1X TBST-1% milk with Odyssey antibody at 1:10,000. Membrane was rinsed again three times in 1X TBST and imaged on the Odyssey.

**Biofilm/Adherence assay**

Cryptococcal adherence assays were performed in a 96-well plate (CLS3631, Corning). Briefly, strains were grown overnight in YPD, diluted to 1 X 10^7^ cells/mL in DMEM, and 100 μl of cell suspension was seeded. Plates were incubated at 37ºC + 5% CO_2_ for 72 hr, washed with PBS, fixed with 4% formaldehyde, blocked with PBS with 1.5% BSA and 0.1% sodium azide, and incubated with anti-GXM primary and secondary antibodies. Plates were imaged on the Odyssey and quantified using ImageStudio software.

Biofilm XTT reduction assays were performed as previously described (13). Briefly, strains were grown overnight in YPD, diluted to 1 X 10^7^ cells/mL in DMEM, and 100 μl of cell suspension was seeded into wells of a microtiter plate. Plates were incubated at 37ºC + 5% CO_2_ for 48 hr, washed with PBS using a microtiter plate washer (405LS, Biotek, Winooski, VT), and 100 μl of XTT/menadione solution (Sigma) was added to each well. The plates were incubated for 3 hr and 75 μl of the supernatant was removed and transferred to a new microtiter plate. Plates were read in a microtiter plate reader at 490 nm.

**Filipin staining**

Staining of membrane sterols was performed as previously described (14, 15). Briefly, strains were grown overnight in YPD and diluted to an OD_600_ of 0.3 and grown for two doublings. Strains were incubated for 5 min with 5 μg/mL of filipin III (Sigma) and washed with PBS. After washing, cells were visualized with a Zeiss DAPI filter set in an Axio Observed inverted microscope. Fluorescence intensity was calculated using Cell Profiler software (https://cellprofiler.org/). Additionally, fluorescent levels were quantified with a fluorescent plate reader (Bio-tek). Fluorescence was measured at the range of 360 to 470 nm wavelength and normalized by OD_600_.

**Virulence assay**

To test virulence in a murine model, strains were cultured overnight in YPD, collected, washed, and diluted to 10^6^ cells/mL in DPBS. Aliquots (50 μL) of the suspension were used to intranasally inoculate groups of five mice (5–6-week-old female A/Jcr mice; Jackson Labs). Animals were closely monitored and sacrificed if they lost >20% relative to peak weight or at the end of the experiment (41 days). Homogenates of lungs, brains, and spleens were plated to determine organ burden.

**REFERENCES**

1. Chang M, Sionov E, Khanal Lamichhane A, Kwon-Chung KJ, Chang YC. 2018. Roles of Three *Cryptococcus neoformans* and *Cryptococcus gattii* Efflux Pump-Coding Genes in Response to Drug Treatment. Antimicrob Agents Chemother 62.

2. Madani G, Lamping E, Lee HJ, Niimi M, Mitra AK, Cannon RD. 2021. Small-Scale Plasma Membrane Preparation for the Analysis of *Candida albicans* Cdr1-mGFPHis. J Vis Exp doi:10.3791/62592.

3. Inglis DO, Skrzypek MS, Liaw E, Moktali V, Sherlock G, Stajich JE. 2014. Literature-based gene curation and proposed genetic nomenclature for *Cryptococcus*. Eukaryot Cell 13:878-83.

4. Fu J, Hettler E, Wickes BL. 2006. Split marker transformation increases homologous integration frequency in *Cryptococcus neoformans*. Fungal Genet Biol 43:200-12.

5. Srikanta D, Yang M, Williams M, Doering TL. 2011. A sensitive high-throughput assay for evaluating host-pathogen interactions in Cryptococcus neoformans infection. PLoS One 6:e22773.

6. Benhamou RI, Bibi M, Steinbuch KB, Engel H, Levin M, Roichman Y, Berman J, Fridman M. 2017. Real-Time Imaging of the Azole Class of Antifungal Drugs in Live *Candida* Cells. ACS Chem Biol 12:1769-1777.

7. Pore VS, Agalave SG, Singh P, Shukla PK, Kumar V, Siddiqi MI. 2015. Design and synthesis of new fluconazole analogues. Org Biomol Chem 13:6551-61.

8. Ivnitski-Steele I, Holmes AR, Lamping E, Monk BC, Cannon RD, Sklar LA. 2009. Identification of Nile red as a fluorescent substrate of the *Candida albicans* ATP-binding cassette transporters Cdr1p and Cdr2p and the major facilitator superfamily transporter Mdr1p. Anal Biochem 394:87-91.

9. Szybalski W, Bryson V. 1952. Genetic studies on microbial cross resistance to toxic agents. I. Cross resistance of Escherichia coli to fifteen antibiotics. J Bacteriol 64:489-99.

10. Santiago-Tirado FH, Peng T, Yang M, Hang HC, Doering TL. 2015. A Single Protein S-acyl Transferase Acts through Diverse Substrates to Determine Cryptococcal Morphology, Stress Tolerance, and Pathogenic Outcome. PLoS Pathog 11:e1004908.

11. Albuquerque P, Nicola AM, Nieves E, Paes HC, Williamson PR, Silva-Pereira I, Casadevall A. 2013. Quorum sensing-mediated, cell density-dependent regulation of growth and virulence in *Cryptococcus neoformans*. mBio 5:e00986-13.

12. Yoneda A, Doering TL. 2008. Regulation of Cryptococcus neoformans capsule size is mediated at the polymer level. Eukaryot Cell 7:546-9.

13. Pierce CG, Uppuluri P, Tristan AR, Wormley FL, Jr., Mowat E, Ramage G, Lopez-Ribot JL. 2008. A simple and reproducible 96-well plate-based method for the formation of fungal biofilms and its application to antifungal susceptibility testing. Nat Protoc 3:1494-500.

14. Altamirano S, Simmons C, Kozubowski L. 2018. Colony and Single Cell Level Analysis of the Heterogeneous Response of *Cryptococcus neoformans* to Fluconazole. Front Cell Infect Microbiol 8:203.

15. Bang S, Kwon H, Hwang HS, Park KD, Kim SU, Bahn YS. 2014. 9-O-butyl-13-(4-isopropylbenzyl)berberine, KR-72, is a potent antifungal agent that inhibits the growth of *Cryptococcus neoformans* by regulating gene expression. PLoS One 9:e109863.
