# Supplementary figures and images for: An Atypical ABC Transporter Is Involved in Antifungal Resistance and Host Interactions in the Pathogenic Fungus Cryptococcus neoformans
Source: mBio. 2022 Jun 21;13(4):e01539-22. doi: 10.1128/mbio.01539-22 (PMC9426558; doi:10.1128/mbio.01539-22)

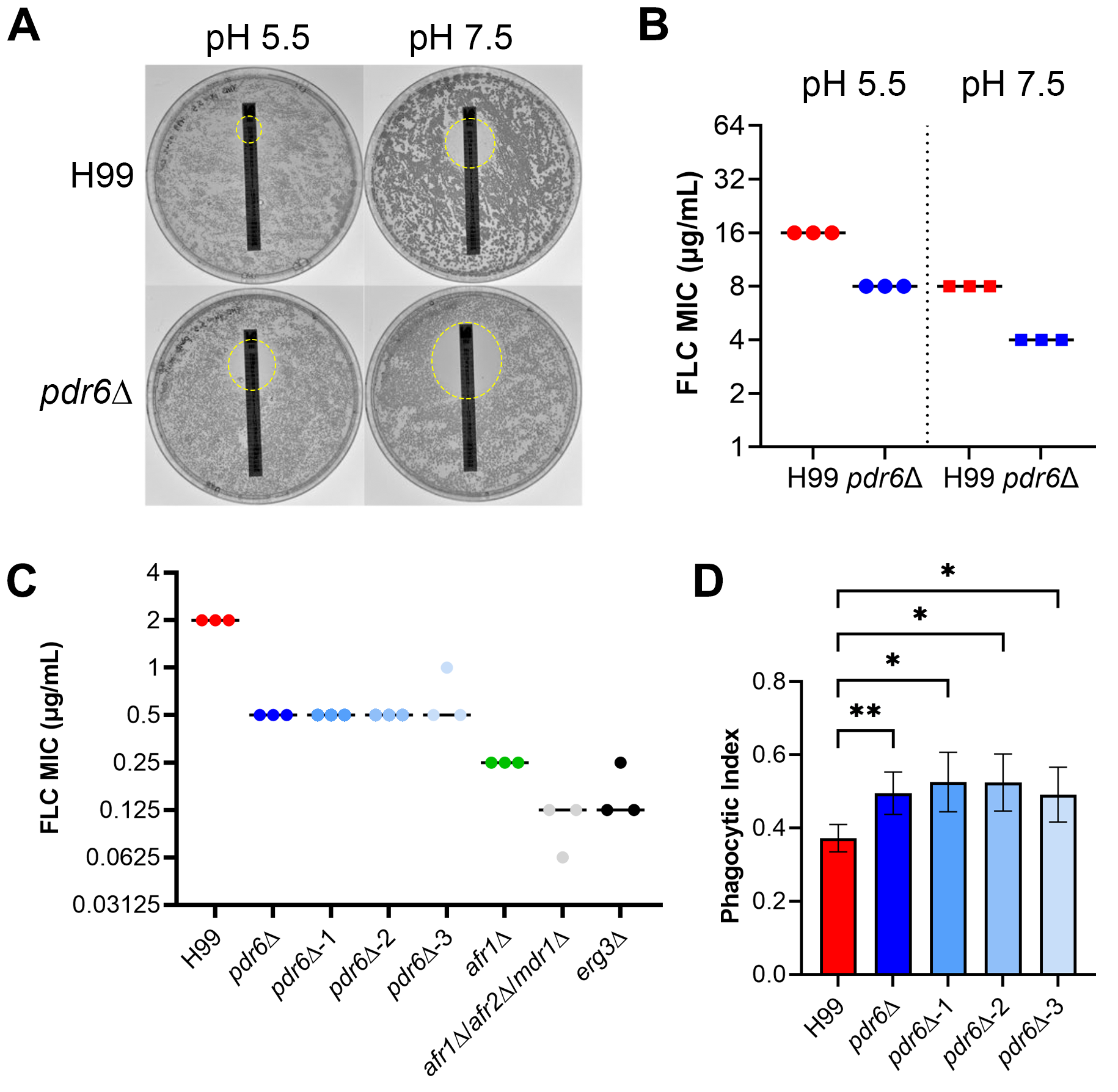

Supplement: FIG S1 [file mbio.01539-22-s0002.tif]

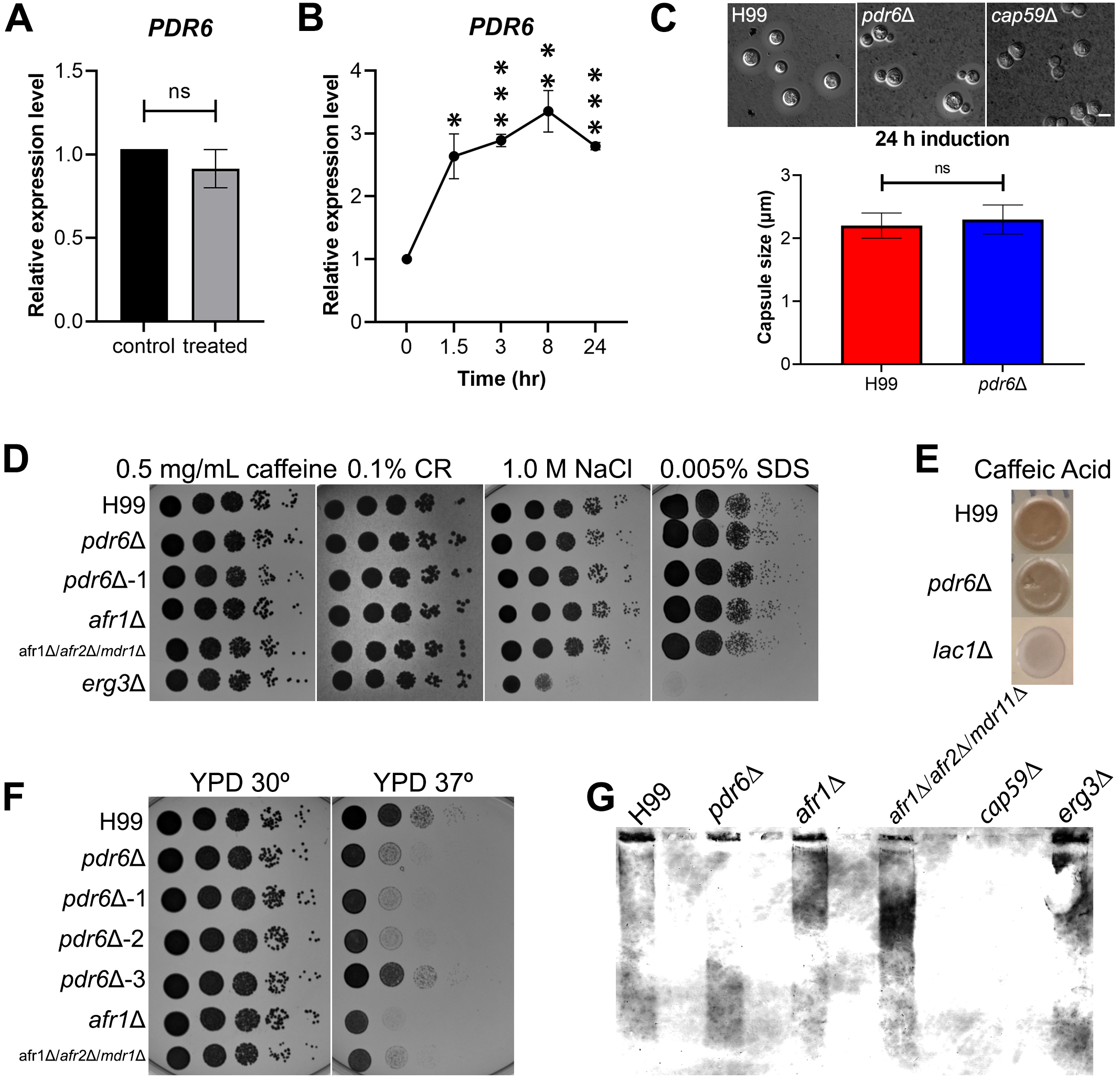

Supplement: FIG S2 [file mbio.01539-22-s0003.tif]
